# Use of the Internet as a Resource for Consumer Health Information: Results of the Second Osteopathic Survey of Health Care in America (OSTEOSURV-II)

**DOI:** 10.2196/jmir.3.4.e31

**Published:** 2001-12-26

**Authors:** John C Licciardone, Peggy Smith-Barbaro, Samuel T Coleridge

**Affiliations:** ^1^Department of Family MedicineTexas College of Osteopathic MedicineUniversity of North Texas Health Science CenterFort Worth TXUSA

**Keywords:** Internet, health care surveys, socioeconomic factors, age factors, family practice

## Abstract

**Background:**

The Internet offers consumers unparalleled opportunities to acquire health information. The emergence of the Internet, rather than more-traditional sources, for obtaining health information is worthy of ongoing surveillance, including identification of the factors associated with using the Internet for this purpose.

**Objectives:**

To measure the prevalence of Internet use as a mechanism for obtaining health information in the United States; to compare such Internet use with newspapers or magazines, radio, and television; and to identify sociodemographic factors associated with using the Internet for acquiring health information.

**Methods:**

Data were acquired from the Second Osteopathic Survey of Health Care in America (OSTEOSURV-II), a national telephone survey using random-digit dialing within the United States during 2000. The target population consisted of adult, noninstitutionalized, household members. As part of the survey, data were collected on: facility with the Internet, sources of health information, and sociodemographic characteristics. Multivariate analysis was used to identify factors associated with acquiring health information on the Internet.

**Results:**

A total of 499 (64% response rate) respondents participated in the survey. With the exception of an overrepresentation of women (66%), respondents were generally similar to national referents. Fifty percent of respondents either strongly agreed or agreed that they felt comfortable using the Internet as a health information resource. The prevalence rates of using the health information sources were: newspapers or magazines, 69%; radio, 30%; television, 56%; and the Internet, 32%. After adjusting for potential confounders, older respondents were more likely than younger respondents to use newspapers or magazines and television to acquire health information, but less likely to use the Internet. Higher education was associated with greater use of newspapers or magazines and the Internet as health information sources. Internet use was lower in rural than urban or suburban areas.

**Conclusions:**

The Internet has already surpassed radio as a source of health information but still lags substantially behind print media and television. Significant barriers to acquiring health information on the Internet remain among persons 60 years of age or older, those with 12 or fewer years of education, and those residing in rural areas. Stronger efforts are needed to ensure access to and facility with the Internet among all segments of the population. This includes user-friendly access for older persons with visual or other functional impairments, providing low-literacy Web sites, and expanding Internet infrastructure to reach all areas of the United States.

## Introduction

The explosion of health information on the Internet is shifting the focus of traditional medical informatics (medical information science) from health professionals to consumers [1]. Consumer informatics is the branch of medical informatics that is concerned with studying consumers' needs for information, providing them with access to health information, and integrating their preferences into medical information systems [1]. This shifting paradigm will have profound effects on the delivery of health care [2,3]. Nevertheless, questions remain about access to the Internet and proclivity to use this medium in health matters [4]. For example, a study of family-practice-clinic patients' intention of using a health information Web site found that those greater than 65 years of age were less likely to do so, even after adjusting for the presence of a home computer and Internet access [5]. The present study was undertaken to measure the prevalence of Internet use in acquiring health information in the United States as compared with other more traditional sources of health information and to identify sociodemographic factors associated with using the Internet for this purpose.

## Methods

We used data from the Second Osteopathic Survey of Health Care in America (OSTEOSURV-II) to measure and determine the factors associated with the use of newspapers or magazines, radio, television, and the Internet as health information sources. The Osteopathic Survey of Health Care in America is a biannual longitudinal survey primarily intended to assess use of osteopathic physicians and public perceptions of osteopathic medicine in the United States. Methodologic details and results of the First Osteopathic Survey of Health Care in America (OSTEOSURV-I) have been published elsewhere [6]. Data from both OSTEOSURV-I and OSTEOSURV-II support the validity and reliability of this research instrument [7] (also J.C.L., unpublished data, 2001)

OSTEOSURV-II was a national telephone survey conducted in 2000 using random-digit dialing. The target population consisted of adult, non-institutionalized, household members. The survey sought to interview 500 respondents, including about 50% who were aware of osteopathic physicians and 25% who had used an osteopathic physician at least once (thus, about one half of those aware of osteopathic physicians would also have used an osteopathic physician at least once). After 361 interviews were completed, awareness and use of osteopathic physicians were 46% and 16%, respectively. Subsequently, random-digit dialing was followed by 2 survey screening items, concerning awareness and use of osteopathic physicians, to increase the percentages of respondents who were aware of or had ever used osteopathic physicians. At the conclusion of the survey, these percentages were 49% and 24%, respectively, thus approaching the initial survey objectives. Also, to increase response during the latter part of the survey, 43 initial nonresponders were subsequently converted into responders by offering a US $20 incentive for participation. All survey procedures were approved by the Institutional Review Board of the University of North Texas Health Science Center.

One section of OSTEOSURV-II was allocated to topics of emerging interest within the American health care environment, including use of the Internet to acquire health information. One survey item stated, "I am comfortable with using the Internet as a health information resource," and was followed by the potential response options: "strongly agree," "agree," "neutral," "disagree," and "strongly disagree." Another item asked, "Do you receive health care information from the following sources?" Responses were solicited for newspapers or magazines, radio, television, and the Internet. All respondents provided sociodemographic information, including age, sex, race or ethnicity, years of education, annual household income, residence (urban or suburban vs. rural), geographic region, health insurance coverage, and general health status as measured by the Medical Outcomes Study Short Form - 36 Health Survey (SF-36) [8].

Descriptive statistics were used to summarize the sociodemographic characteristics of respondents and their sources of health information. Contingency tables and the **c** ^2^ test were used to identify statistical associations between sociodemographic characteristics and use of the various sources of health information. Multiple logistic regression was then used to compute the odds ratios (ORs) and 95% confidence intervals (CIs) for each source of health information while simultaneously adjusting for age, sex, race or ethnicity, education, residence, and geographic region. Statistical analyses were performed using the SYSTAT software package (SPSS Inc, Chicago, IL) and all hypotheses were tested at the .05 level of statistical significance.

## Results

A total of 499 (64% response rate) respondents participated in the survey. Of these, 329 (66%) respondents were women. Otherwise, as shown in Table 1, respondents were generally similar to national referents based on information from the US Census Bureau [9] and normative standards for the SF-36 [8].

Fifty percent of respondents either strongly agreed or agreed that they felt comfortable using the Internet as a health information resource. Persons 60 years of age or older (**c** ^2^_2_=28.3; *P*<.001) and those with 12 or fewer years of education (**c** ^2^_2_=22.1; *P*<.001) reported less comfort with the Internet. The prevalence rates of using the health information sources were: newspapers or magazines, 69%; radio, 30%; television, 56%; and the Internet, 32%.

**Table 1:** Sociodemographic Characteristics of OSTEOSURV-II Respondents*

	Survey Respondents (n=499)		National Referents †
**Characteristic**	No.	%	%
**Age (years)**, mean (SD)	46.3 (16.5)		44.9 (16.1)
**18-29**	90	18	
**30-39**	100	20	
**40-49**	115	23	
**50-59**	72	14	
**60-69**	64	13	
**> 70**	57	11	
**Sex**			
**Men**	170	34	48
**Women**	329	66	52
**Race**‡			
**White**	405	86	84
**Black**	40	8	12
**Asian/Pacific Islander**	18	4	4
**American Indian/ Native American**	9	2	1
**Geographic region**			
**Northeast**	88	18	19
**Midwest**	132	27	24
**South**	179	36	36
**West**	96	19	21
**Education, years**			
**<12**	57	11	17
**12**	121	24	33
**13-15**	172	35	25
**16**	86	17	17
**> 17** >17	62	12	8
**Residence**§			
**Urban/suburban**	310	63	75
**Rural**	179	37	25
**Annual household income, median, $**	38,318		38,885
**<15,000**	58	13	
**15,001-25,000**	75	16	
**25,001-40,000**	107	24	
**40,001-60,000**	83	18	
**> 60,001** >60,001	132	29	
**Health insurance coverage**			
**No**	49	10	13
**Yes**	449	90	87
**General health perceptions**#			
MOS SF-36 score, mean (SD)	71.6 (21.1)		72.0 (20.3)

^*^ Data are presented as number or percentage unless otherwise indicated. Totals may not equal 499 because of item nonresponse and may differ from 100% because of rounding. OSTEOSURV-II denotes the Second Osteopathic Survey of Health Care in America; MOS SF-36, Medical Outcomes Study Short - Form-36 Health Survey.

^†^ Referent characteristics were based on information derived from the US Bureau of the Census [9] except for general health perceptions. For variables that were categorized differently by the Bureau of the Census and OSTEOSURV-II, only the mean (SD) or median were compared using methods for grouped data.

^‡^ A total of 13 (3%) respondents who described themselves as Hispanic are not included in the table because persons of Hispanic origin may be of any race.

^§^ Referents are categorized as urban or rural.

^#^ Referents for this characteristic were selected from the general United States population [8]. SD = standard deviation

**Table 2:** Prevalence Rates of and Multivariate Factors Associated with Use of Health Information Sources*

	**Newspapers/Magazines**				Radio				Television				Internet			
**Characteristic**†	**PR**	**OR**	**95% CI**	**P**	**PR**	**OR**	**95% CI**	**P**	**PR**	**OR**	**95% CI**	**P**	**PR**	**OR**	**95% CI**	**P**
**Age, years**																
18-39	60	1.00			31	1.00			48	1.00			36	1.00		
40-59	78	1.98	1.22 - 3.22	.01	34	1.16	0.74 - 1.83	.52	61	1.65	1.07 - 2.54	.02	39	0.93	0.59 - 1.46	.76
>60	70	1.94	1.11 - 3.39	.02	23	0.81	0.46 - 1.41	.45	58	1.71	1.03 - 2.83	.04	14	0.30	0.16 - 0.58	<.001
**Sex**																
Men	63	1.00			29	1.00			51	1.00			33	1.00		
Women	73	1.57	1.01 - 2.44	.04	30	1.05	0.68 - 1.60	.83	58	1.27	0.85 - 1.88	.24	31	1.01	0.65 - 1.57	.95
**Race/ethnicity**‡																
White	71	1.00			30	1.00			55	1.00			32	1.00		
Non-White	64	0.79	0.44 - 1.39	.41	30	1.01	0.58 - 1.77	.96	61	1.45	0.85 - 2.47	.17	33	0.73	0.41 - 1.28	.27
**Education, years**																
<12	54	1.00			26	1.00			51	1.00			16	1.00		
13-15	75	2.70	1.64 - 4.43	<.001	35	1.47	0.91 - 2.39	.12	56	1.32	0.84 - 2.07	.23	36	2.48	1.45 - 4.24	.001
>16	81	3.19	1.84 - 5.50	<.001	29	1.08	0.64 - 1.82	.78	61	1.49	0.92 - 2.41	.10	45	3.25	1.87 - 5.65	<.001
**Residence**																
Urban/suburban	73	1.00			29	1.00			59	1.00			36	1.00		
Rural	63	0.66	0.43 - 1.03	.07	33	1.18	0.77 - 1.80	.44	52	0.81	0.54 - 1.21	.30	26	0.59	0.38 - 0.93	.02
**Geographic region**																
Northeast	80	1.00			36	1.00			60	1.00			38	1.00		
Midwest	70	0.66	0.34 - 1.30	.23	33	0.83	0.46 - 1.49	.52	53	0.81	0.46 - 1.44	.48	25	0.63	0.33 - 1.19	.15
South	67	0.64	0.34 - 1.22	.17	29	0.64	0.36 - 1.13	.12	58	1.05	0.61 - 1.82	.86	32	1.00	0.56 - 1.81	.99
West	65	0.44	0.22 - 0.89	.02	24	0.54	0.28 - 1.04	.06	52	0.71	0.38 - 1.31	.27	34	0.92	0.48 - 1.78	.81

^*^ The various analyses included 470 (94%) to 473 (95%) respondents who provided complete data. Prevalence rate is reported as a percentage.

^†^ PR = prevalence rate, OR = odds ratio, CI = confidence interval.

^‡^ Hispanics were included in the non-White category for these analyses.

Specific prevalence rates and multivariate ORs and CIs for each health information source are presented in Table 2. After adjusting for potential confounders, older respondents were more likely than younger respondents to use newspapers or magazines and television to acquire health information, but less likely to use the Internet. Women were more likely than men to acquire health information from newspapers or magazines. Higher education was associated with greater use of newspapers or magazines and the Internet as health information sources. Internet use was lower in rural than urban or suburban areas and newspaper or magazine use was lower in the West than in the Northeast.

## Discussion

Our survey indicates that one half of the American adult population are comfortable using the Internet as a health information resource and that about one third actually use the Internet to acquire health information. These findings regarding overall Internet use are generally consistent with other studies in this area. The Pew Internet & American Life Project conducted a large national telephone survey using random-digit dialing during the same time period as our survey and estimated that 52 million American adults (55% of those with Internet access) have used the Internet to acquire health information [10]. Based on an estimated 205 million adults in the United States in 2000 [9], this indicates that 25% of American adults used the Internet to obtain health information. A random telephone survey of California households in 1998 found that 19% had used the Internet to acquire health information within the past year, compared with 31% who used newspapers or magazines [11]. Parenthetically, that Californians also identified newspapers and magazines as the most distrusted sources of health information [11], also supports our finding of a decreased use of newspapers or magazines for obtaining health information in the western United States. A study of patients who had undergone coronary artery bypass grafting found that 22% had used the Internet to acquire health information [12]. It has been recently reported that 22% of patients in Japan use the Internet to obtain health information [13].

The Pew Internet & American Life Project concluded that health information seekers on the Internet are proportionately more middle-aged than very young or old and more likely to be women than men, but that there are no major racial, ethnic, or income effects [10]. The only notable discrepancy between these results and our findings involves health information seeking on the Internet among men and women. We found no sex differences in this regard. The overrepresentation of women in our survey may be a potential explanation for this discrepancy. It is reasonable to speculate that the overrepresentation of women was attributable to respondents who were unemployed outside the home. Such unemployment would have precluded Internet use at the workplace, which is known to be a common site for Internet access [11].

The Internet has already surpassed radio as a source of health information but still lags substantially behind print media and television. Significant barriers to acquiring health information on the Internet remain among persons 60 years of age or older, those with 12 or fewer years of education, and those residing in rural areas.

There are 3 potential limitations of our survey that should be mentioned. First, the survey was conducted over one year ago and it is possible that Internet access and use may have increased since then. Second, as noted above, the survey included a disproportionately large representation of women (66%). This is a common finding in many surveys despite special techniques to minimize this problem [14,15]. Third, we used 2 screening items during the latter part of the survey to select a greater percentage of respondents who were either patients or aware of osteopathic physicians. This screening may have also selected respondents with somewhat better education than referents, as evidenced by the greater percentage of respondents with at least some college education (64% vs. 50%). Although these 3 factors may have biased our prevalence estimates to some degree, it is unlikely that they materially affected the survey findings. The use of multivariate modeling to adjust for potential confounders, such as sex and education, further attenuated any biases that may have been introduced by the large percentage of women who responded and by screening for use or awareness of osteopathic physicians.

Our findings have important implications, because consumer informatics is rapidly evolving with a public health focus that seeks to provide a greater emphasis on prevention and self care [1]. Stronger efforts are needed to ensure access to and facility with the Internet among all segments of the population. This includes user-friendly access for older persons with visual or other functional impairments, providing low-literacy Web sites, and expanding Internet infrastructure to reach all areas of the United States.

## Abbreviations

CI: Confidence Interval
OR: Odds Ratio
PR: Prevalence Rate
SD: Standard Deviation
